# Boar-to-Boar Variations in Quality Characteristics of Sperm from Different Ejaculates Following Freezing–Thawing

**DOI:** 10.3390/cells14030212

**Published:** 2025-02-02

**Authors:** Leyland Fraser, Łukasz Zasiadczyk, Marzena Mogielnicka-Brzozowska

**Affiliations:** Department of Animal Biochemistry and Biotechnology, Faculty of Animal Bioengineering, University of Warmia and Mazury in Olsztyn, 10-719 Olsztyn, Poland; lukasz.zasiadczyk@uwm.edu.pl (Ł.Z.); mmog@uwm.edu.pl (M.M.-B.)

**Keywords:** boar, sperm-rich fractions, ejaculates, cryopreservation, freezability

## Abstract

The main objective of this study was to investigate boar-to-boar variations in the quality characteristics of sperm from the sperm-rich fractions (SRFs) and whole ejaculates (WEs) following freezing–thawing. Several sperm attributes, such as motility patterns analyzed by the computer-assisted sperm analysis (CASA) system, mitochondrial function, membrane integrity, and DNA fragmentation were used to compare the cryo-survival of sperm from SRFs and WEs from boars with good and poor semen freezability (GSF and PSF, respectively). In this study, boars with post-thaw total motility (TMOT) more than 30% (>30%) were classified as having GSF, while those with post-thaw TMOT less than 30% (<30%) were classified as having PSF. Principal component analysis 1 (PCA1), which is the main component of the sample variation, explained approximately 75% of the variance between the GSF and PSF groups, reaffirming the reliability of post-thaw TMOT as a reliable criterion used to classify the animals. Most of the post-thaw sperm parameters of the SRFs and WEs were positively correlated. Furthermore, scatter plot analyses show stronger relationships between the analyzed post-thaw parameters of the frozen–thawed (FT) sperm of SRFs than those of WEs. Individual boar variations or the sperm source had marked effects on the quality characteristics of FT sperm. The higher TMOT, velocity straight line (VSL), and velocity average path (VAP) of FT sperm were more enhanced in the SRFs compared with the WEs of the PSF group. Furthermore, the mitochondrial function, membrane integrity, and DNA fragmentation of FT sperm were markedly higher in the SRFs than in the WEs, particularly for the poor freezability boars. We suggest that the freezability potential of sperm of the GSF group does not differ significantly between the SRFs and WEs, reaffirming that boar variability is an important factor that affects the cryo-survival of sperm.

## 1. Introduction

The cryopreservation of semen allows the preservation of valuable genetic material [1,2], and it has been suggested to be one of the most promising biotechnological tools that could have a great impact on the global swine industry [3]. There is evidence that semen cryopreservation induces different structural and biochemical alterations in the membrane of spermatozoa, resulting in their reduced fertilizing ability [1,3,4]. Moreover, the quality of post-thaw semen is dependent on several factors, including the sperm source and individual variability [5,6,7,8]. Besides damage induced by reactive oxygen species (ROS) [3,9,10], cryopreservation affects the functions of the sperm mitochondrial proteins associated with different biochemical process, including phosphorylation, thus compromising the metabolic activity of frozen–thawed (FT) sperm [11].

Differences in the freezability of boar semen still represent a challenge regarding the improvement of the cryopreservation procedure and the large-scale application of FT semen in artificial insemination (AI) techniques [2,3,4]. Individual variability among boars has been shown to exert a notable effect on sperm cryo-tolerance [6,7,8]. This variability has been suggested to be due to genetic factors [5,12], and recent studies have confirmed that sperm-related gene expression is a potential marker for the freezability of boar semen [13,14,15].

Notably, the biochemical composition of boar seminal plasma (SP) varies significantly among ejaculates from the same boar [16]. This factor contributes to varying effects on the post-thaw quality of sperm as well as their fertilizing ability [17,18,19,20]. Boar ejaculate comprises three main fractions—the pre-sperm fraction (PSF); the sperm-rich fraction (SRF); and the post-SRF, which contributes the bulk of the SP originating from the testis, epididymis, and accessory sex glands [16]. There are still conflicting reports about the effects of boar SP on sperm functions following cryopreservation. Accumulating evidence has been shown that the presence of SP fractions of boar ejaculates could modulate the functions of FT sperm [17,18,19,21,22]. Other studies have reported on the beneficial effects of a pre-freeze (PF) holding period of sperm in SP from different fractions of boar ejaculates on cryo-tolerance [23,24,25]. There are still variable results regarding the effects of SP from different fractions of boar ejaculates on sperm cryo-survival [26,27]. Recently, it has been reported that the inclusion of the whole ejaculate (WE, which includes SRF + post-SRF) in AI doses does not compromise liquid-stored semen quality, fertility, prolificacy, and offspring performance [28].

The aim of this study was to investigate the individual boar variability in the quality characteristics of sperm from sperm-rich fractions (SRFs) and whole ejaculates (WEs) following freezing–thawing. We used several sperm attributes (motility patterns analyzed by the computer-assisted sperm analysis (CASA) system, mitochondrial function, membrane integrity, and DNA fragmentation) to compare the post-thaw quality characteristics of sperm from the SRFs and WEs from the good and poor semen freezability (GSF and PSF, respectively) groups.

## 2. Materials and Methods

### 2.1. Chemicals and Media

Chemicals were bought from Sigma Chemical Company (St. Louis, MO, USA), unless otherwise stated.

### 2.2. Animals

Ten Polish large white (PLW) boars (average aged two years), stationed at the Cryopreservation Laboratory, Department of Animal Biochemistry and Biotechnology, Faculty of Animal Bioengineering, were used in this study. The SRFs (approximately 20 mL) were collected once weekly from the boars followed by the collections of the WEs (SRFs + post SRFs) from the same boars. All procedures involving animals were carried out in accordance with the guidelines outlined by the Polish Local Ethics Committee, Olsztyn, Poland (number: 90/2012 and number: 24/2015). Approval from the Local Ethics Committee for semen processing (collection procedure) has not been required since 15 January 2015.

### 2.3. Semen Collections

The SRFs were collected once weekly from the boars followed by the collections of the WEs. The SRFs and WEs were collected from each boar during the autumn–winter period. A total of five or seven SRFs or WEs were collected from each boar using the gloved-hand technique [21]. Water was available ad libitum. Fresh, PF semen samples with more than 70% total motility (TMOT) of sperm and 85% morphologically normal sperm [25,29] were used in the study. Sperm concentration was determined cytometrically, using a Bürker counting chamber (Equimed-Medical Instruments, Cracow, Poland) [30].

### 2.4. Semen Cryopreservation Procedure

Semen samples were frozen using a standard cryopreservation protocol [21,25,30]. The SRFs were extended (1:4) with Beltsville Thawing Solution (BTS), while the WEs were extended (1:1) with the same extender prior to centrifugation (800× *g*, 10 min at room temperature). The recovered sperm pellets (750 × 10^6^ sperm/mL) were re-suspended in an extender (Extender I) containing 11% lactose and lipoprotein fractions of ostrich egg yolk, (LPFo), and they were cooled for a 2 h period at 5 °C. The cooled semen was further extended (2:1) with a freezing extender, consisting of 89.5 mL extender I, 9 mL glycerol, and 1.5 mL Orvus Es Paste (OEP), before being packaged into sterilized aluminum cryotubes (Factory of Medical Materials, Polfa, S.A. Boleslawiec, Poland). The samples (50 × 10^6^ sperm/mL) were frozen in a programmable controlled-rate freezer (Ice Cube 1810, SY-LAB, Purkersdorf, Austria) [30]. The frozen samples were thawed in a water bath for 60 s at 50 °C extended (1:10) in BTS and held in a water bath for 15 min at 37 °C for post-thaw analysis.

### 2.5. Semen Quality Assessment

Sperm quality characteristic assessments were performed for the fresh, PF and FT semen.

#### 2.5.1. Motility Parameters Analyzed by the CASA System

The computer-assisted sperm analysis (CASA) system (HTR-IVOS 12.3, Hamilton Thorne Biosciences, Beverly, MA, USA) was used to assess the sperm motility parameters. Sperm samples (5 mL) were placed on a Makler chamber (Counting Chamber Makler, Sefi-Medical Instruments, Ltd., Haifa, Israel), pre-warmed at 38 °C, and were analyzed, using a negative phase contrast, 10× objective and magnification of 1.92. The sperm parameters assessed by the CASA system included TMOT (%), progressive motility (PMOT, %), velocity straight line (VSL, μm/s), velocity average path (VAP, μm/s), velocity curvilinear (VCL, μm/s), straightness (STR, ratio of VSL/VAP × 100, %), linearity (LIN, ratio of VSL/VCL × 100, %), amplitude of lateral head displacement (ALH, μm), and beat cross frequency (BCF, Hz).

The CASA sperm motility parameters were measured with the following settings: frame required, 45; frame rate, 60 Hz; minimum cell contrast, 46; minimum cell size, 7 pixels; straightness threshold, 45%; velocity average path (VAP, micrometers per second) threshold, 45 mm/s; low VAP cut-off, 20 μm/s; and low straight line velocity (VSL, micrometers per second) cut-off, 5.0 μm/s [13,24].

#### 2.5.2. Mitochondrial Function

The percentage of sperm with functional mitochondrial membrane potential (MMP) was evaluated with the fluorescent lipophilic cation JC-1 and propidium iodide (PI) fluorescent dyes [31]. Briefly, aliquots of sperm samples were diluted in a HEPES-buffered solution, stained with JC-1 (1 mg JC-1/mL DMSO) for 15 min at 37 °C and then stained with a PI solution (2.4 µM PI in Tyrode’s salt solution) for 10 min at 37 °C, before being examined at 600× magnification using a fluorescence microscope (Olympus CH 30, Tokyo, Japan). A minimum of 100 sperm cells per slide were examined, and sperm that exhibited orange-red fluorescence in the midpiece region were considered as viable sperm cells with high MMP. Slides were analyzed in duplicate.

#### 2.5.3. Plasma Membrane and Acrosome Integrity

The Live/Dead Sperm Viability Kit was used to assess the percentage of sperm with plasma membrane integrity (PMI), Briefly, aliquots of sperm samples were incubated with 1 mM SYBR-14 solution in HEPES-BSA (pH 7.4) and a PI solution for 10 min at 37 °C. A minimum of 100 cells per slide were examined at ×600 magnification under a fluorescence microscope (Olympus CH 30), and two slides were evaluated per sample [32]. The percentage of sperm with normal apical ridge (NAR) acrosome integrity was assessed according to a previously described method [25,29]. Aliquots of Giemsa-stained sperm samples were examined under a bright light microscope, equipped with oil-immersion lens at ×1000 magnification (Olympus BX 41, Olympus, Tokyo, Japan). A minimum of 100 sperm cells per slide were examined, with two slides per sample.

#### 2.5.4. DNA Fragmentation

The Comet assay was used to assess sperm DNA fragmentation [21,30]. Briefly, sperm samples (10 × 10^6^ sperm/mL) were lysed before being subjected to electrophoresis. A minimum of 200 sperm cells per slide were examined in random fields at 400× magnification under a fluorescence microscope (Olympus BX 41, Tokyo, Japan). Aliquots of sperm samples were classified as non-fragmented DNA (undamaged) and fragmented DNA (damaged) sperm cells.

### 2.6. Clustering and Correlation Analyses

Clustering analysis was performed to visualize boars of the GSF and PSF groups, using the SRplot (https://www.bioinformatics.com.cn/plot_basic_PCA_plot_034_en, accessed on 21 August 2024) [33]. Clustering analysis was performed to visualize boars of the freezability groups according to the post-thaw TMOT of sperm from the SRFs and WEs. Also, correlation coefficient matrices of the analyzed quality characteristics of FT sperm from the SRFs and WEs were plotted using the SRplot [33].

### 2.7. Statistical Analysis

The Shapiro–Wilk W-test was used to analyze the normality of the data distribution. Data of the fresh, PF or post-thaw semen (VSL, VAP, VCL, ALH, and BCF) were log10-transferred to meet the normality assumption and were examined with the General Linear Model (GLM) procedure from Statistica software package, version 13.3 (Statistica software package, version 13.3, TIBCO Software Inc. 2017; Statistica, CA, USA; StatSoft Polska, Kraków, Poland) and the IBM SPSS Statistics software package (IBM Corp. released 2020. IBM SPSS Statistics for Windows, version 27.0, IBM Corp., Armonk, NY, USA). The post-thaw TMOT of FT sperm from the SRFs and WEs was used to classify boars in the GSF and PSF groups. The mean TMOT of FT sperm of more than 30% (>30%) was used as a criterion to classify boars as having GSF, whereas boars with post-thaw sperm TMOT less than 30% (<30%) were considered as having PSF [13,34,35]. The assessment of post-thaw semen quality showed that five boars (Boar nos. 1 to 2, Boar nos. 4 to 5, and Boar no. 7) exhibited GSF, while the other five boars, (Boar no. 3, Boar no. 6, and Boar nos. 8 to 10) were considered as having PSF.

A two-way analysis of variance (ANOVA) was performed to evaluate the data. A 10 × 2 factorial design was performed to determine if boar and sperm source (SRF and WE), or their interactions, affected the quality characteristics of the fresh, PF semen. A 5 × 2 factorial design was performed to determine if boars from either the GSP group or PSF group and sperm source, or their interactions, affected post-thaw sperm quality. Scatterplots with correlation coefficients were plotted to illustrate the relationships of sperm TMOT of the SRFs or WEs with most of the sperm parameters following freezing–thawing. All results are expressed as the mean ± standard error of the mean (S.E.M). Significant differences among groups within the freezability group were compared using the Tukey HSD post hoc test. The values were considered to differ significantly at *p* < 0.05.

## 3. Results

### 3.1. Clustering Analysis

**Figure 1:**
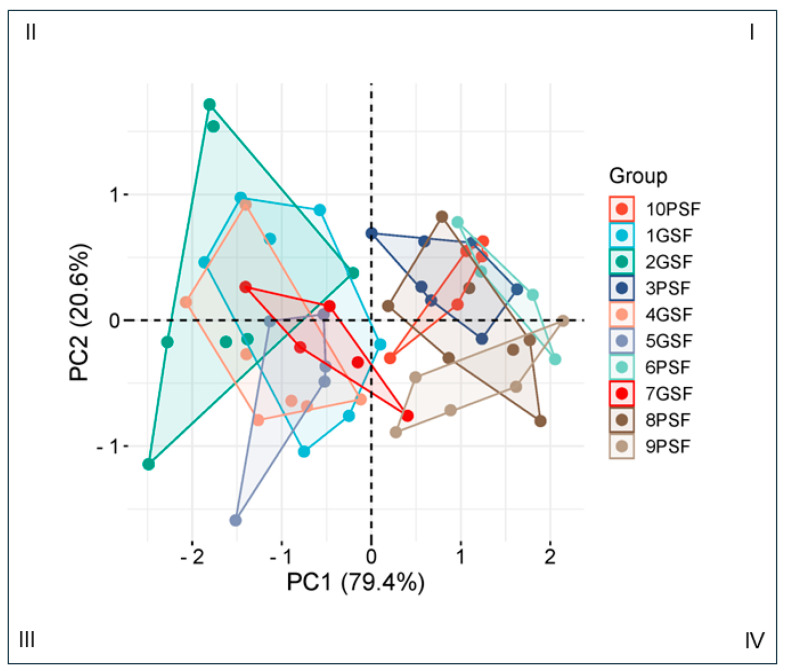
Principal component analysis (PCA) of total motility (TMOT) of frozen–thawed (FT) sperm from boars with good semen freezability (GSF) and poor semen freezability (PSF). Each color represents an individual sample from the sperm-rich fraction (SRF) and whole ejaculate (WE). The quadrants of the plot are represented by nos. I to IV.

The clustering analysis showed more than 95% variations in post-thaw sperm total motility (TMOT) between boars of the GSF and PSF groups (Figure 1). Principal component analysis 1 (PCA1) confirmed approximately 75% of the variances, while PCA2 confirmed approximately 21% of the variances between the GSF and PSF groups. Boars of the GSF group (Boar nos. 1 to 2, Boar nos. 4 to 5, and Boar no. 7) were clustered mainly in quadrants II and III, while those of the PSF group (Boar no. 3, Boar no. 6, and Boar nos. 8 to 10) were clustered in quadrants I and IV (Figure 1).

### 3.2. Assessment of Fresh, PF Semen Quality

ANOVA results that neither individual boar variations nor sperm source had a significant effect (*p* > 0.05) on the fresh, PF semen quality (Supplementary Table S1). Likewise, the boar × sperm source interaction did not significantly affect (*p* > 0.05) the fresh, PF semen quality (Supplementary Table S1). No marked effects (*p* > 0.05) were observed for the analyzed sperm quality characteristics (Supplementary Table S2).

### 3.3. Assessment of Post-Thaw Semen Quality

#### 3.3.1. ANOVA Effects on FT Sperm of the GSF and PSF Groups

Post-thaw TMOT, VSL, LIN, ALH, and BCF, analyzed by the CASA system, were significantly affected (*p* < 0.05) either by individual boar variations or the sperm source (SRFs and WEs) in the GSF group (Table 1). It was observed that only individual boar variations significantly affected (*p* < 0.05) the velocity average path (VAP), while the sperm source had significant effects (*p* < 0.05) on the PMOT, STR, MMP, and DNA fragmentation of FT sperm from the GSF group (Table 1). In addition, the boar × sperm source interaction had significant effects (*p* < 0.05) only on the post-thaw LIN and BCF values of the GSF group (Table 1). Also, the VCL values, PMI, and NAR acrosome integrity of FT sperm of the GSF group were not affected (*p* > 0.05) by individual boar variations or the sperm source (Table 1).

Post-thaw analysis showed that the CASA-analyzed velocity parameters (VSL, VAP, and VCL), NAR acrosome integrity, and DNA fragmentation were significantly affected (*p* < 0.05) either by individual boar variations or the sperm source of the PSF group (Table 2). Individual boar variations had significant effects (*p* < 0.05) on the post-thaw LIN and BCF values and PMI, while the sperm source had marked effects (*p* < 0.05) on the post-thaw TMOT, ALH values, and MMP of the poor freezability group (Table 2). Most of the parameters of the post-thaw sperm quality characteristics (TMOT, PMOT, VSL, BCF, PMI, NAR acrosome integrity, and DNA fragmentation) were significantly affected (*p* < 0.05) by the boar × sperm source interaction (Table 2). Furthermore, post-thaw STR values were not affected (*p* > 0.05) by individual boar variations or the sperm source (Table 2).

#### 3.3.2. Evaluation of CASA-Analyzed Sperm Parameters

**Figure 2:**
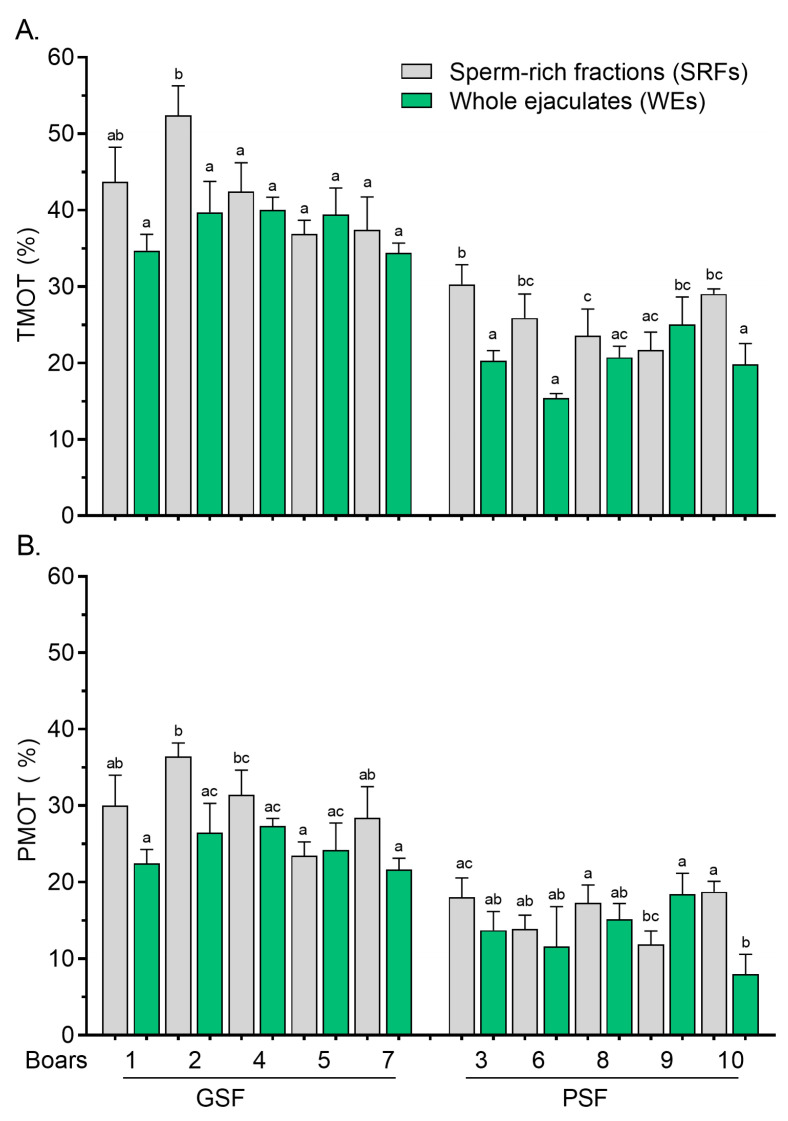
Total motility, TMOT, (**A**) and progressive motility, PMOT, (**B**) of frozen–thawed (FT) boar sperm. Within the freezability group, values with different letters (a, b, and c) are significant at *p* < 0.05. GSF—good semen freezability; PSF—poor semen freezability.

The TMOT of FT sperm was significantly reduced (*p* < 0.05) in the WEs compared with the SRFs, particularly for at least three boars (Boar no. 3, Boar no. 6, and Boar no. 10) of the PSF group (Figure 2A).

**Figure 3:**
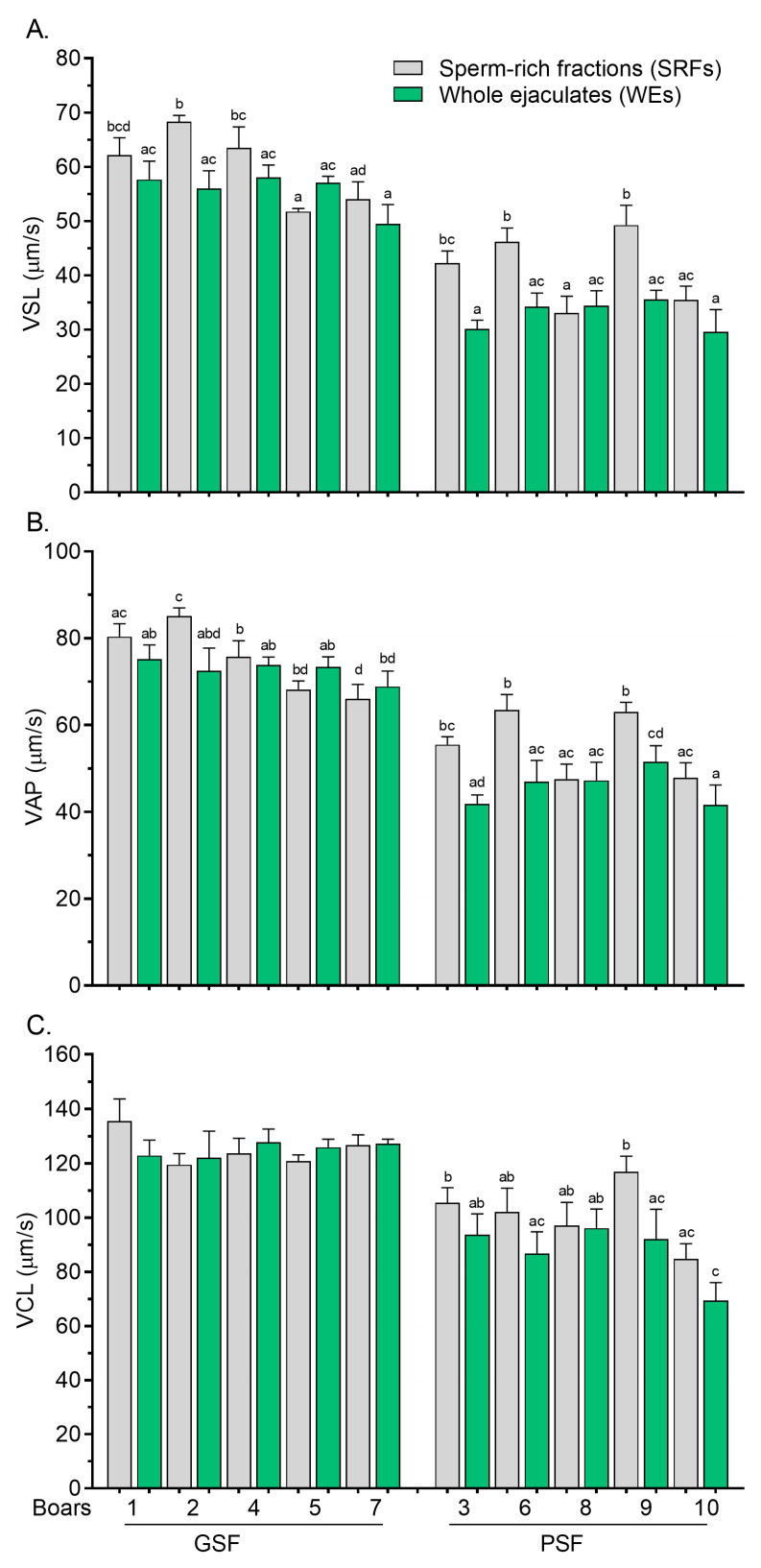
Velocity straight line, VSL, (**A**) velocity average path, VAP, (**B**) and velocity curvilinear, VCL, (**C**) of frozen–thawed (FT) boar sperm. Within the freezability group, values with different letters (a, b, c, and d) are significant at *p* < 0.05. GSF—good semen freezability; PSF—poor semen freezability.

Significantly lower (*p* < 0.05) variations in the PMOT of FT sperm between the SRFs and WEs were less pronounced among the boars, regardless of the freezability group (Figure 2B). Similarly to post-thaw, the TMOT, VSL, and VAP values of FT sperm were significantly reduced (*p* < 0.05) in the WEs compared with the SRFs, and they were observed in three boars of the PSF group, Boar no. 3, Boar no. 6, and Boar no. 9 (Figure 3A and Figure 3B, respectively). In addition, variations in post-thaw VCL values between the SRFs and WEs were more marked (*p* < 0.05) in Boar no. 9 of the PSF group (Figure 3C).

**Figure 4:**
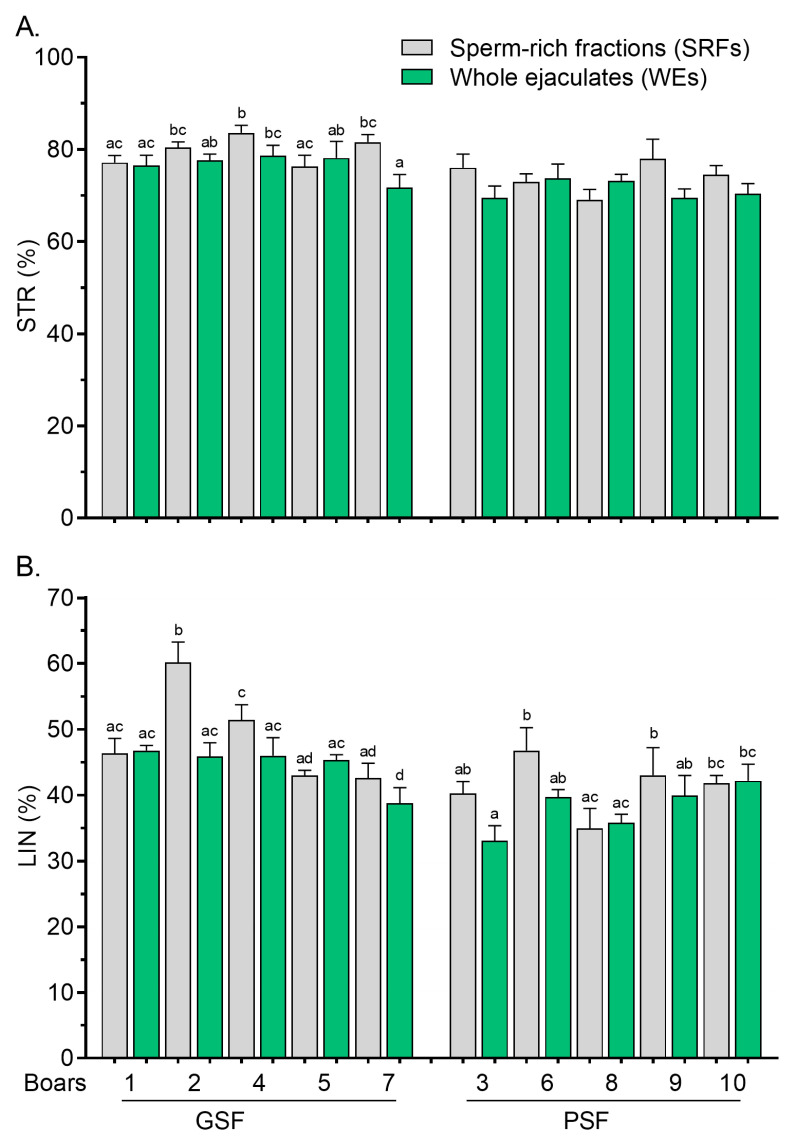
Straightness, STR, (**A**) and linearity, LIN, (**B**) of frozen–thawed (FT) boar sperm. Within the freezability group, values with different letters (a, b, c, and d) are significant at *p* < 0.05. GSF—good semen freezability; PSF—poor semen freezability.

**Figure 5:**
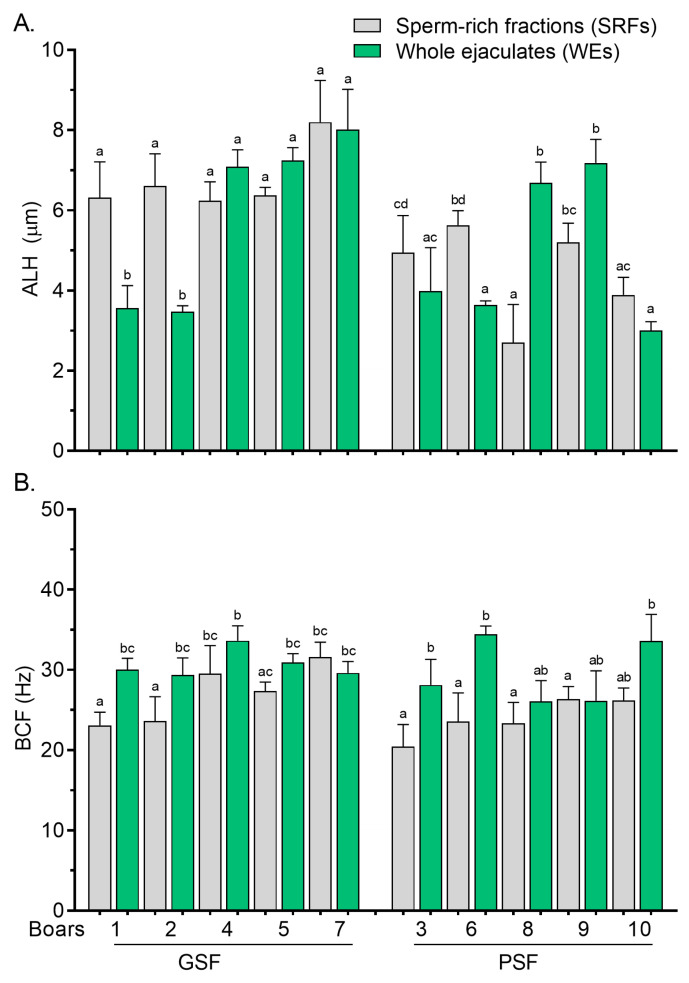
Amplitude of lateral head displacement, ALH, (**A**) and beat cross frequency, BCF, (**B**) of frozen–thawed (FT) boar sperm. Within the freezability group, values with different letters (a, b, c, and d) are significant at *p* < 0.05. GSF—good semen freezability; PSF—poor semen freezability.

Differences in post-thaw STR values between the SRFs and WEs were less marked among the boars of the GSF group (Figure 4A), while there were no significant variations (*p* > 0.05) in the STR values of the PSF group following thawing (Figure 4A). Variations in post-thaw LIN values between the SRFs and WEs were more marked in the GSF group (Figure 4B). Wide variations were observed in post-thaw ALH and BCF values, particularly in the GSF group (Figure 5A and Figure 5B, respectively).

Post-thaw ALH values were significantly higher (*p* < 0.05) in SRFs from Boar nos. 1 to 2 of the GSF group (Figure 5A). In contrast, post-thaw BCF values were significantly reduced (*p* < 0.05) in the SRFs of Boar nos. 1 to 2 of the GSF compared with the WEs (Figure 5B).

#### 3.3.3. Evaluation of Sperm Mitochondrial Function and Membrane Integrity

**Figure 6:**
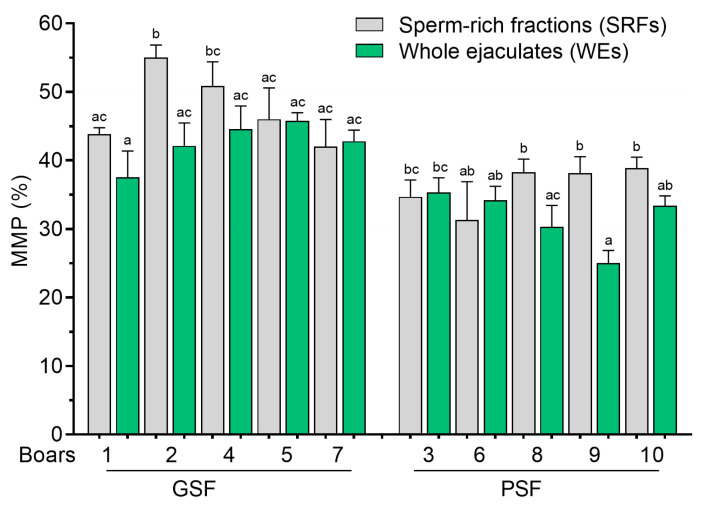
Mitochondrial membrane potential (MMP) of frozen–thawed (FT) boar sperm. Within the freezability group, values with different letters (a, b, and c) are significant at *p* < 0.05. GSF—good semen freezability; PSF—poor semen freezability.

Wide variations in post-thaw sperm MMP were observed between the SRFs and WEs, regardless of the freezability group (Figure 6). It was observed that, in at least two boars of the PSF group (Boar nos. 8 to 9), post-thaw MMP was significantly higher (*p* < 0.05) in the SRFs than in the WEs (Figure 6).

**Figure 7:**
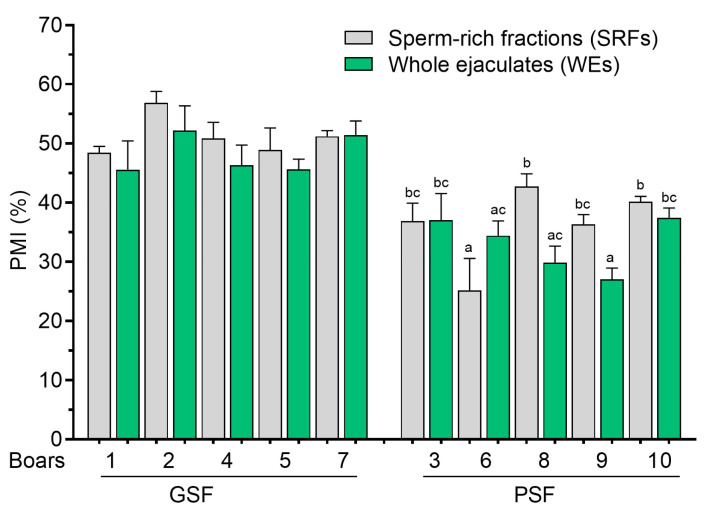
Plasma membrane integrity (PMI) of frozen–thawed (FT) boar sperm. Within the freezability group, values with different letters (a, b, and c) are significant at *p* < 0.05. GSF—good semen freezability; PSF—poor semen freezability.

**Figure 8:**
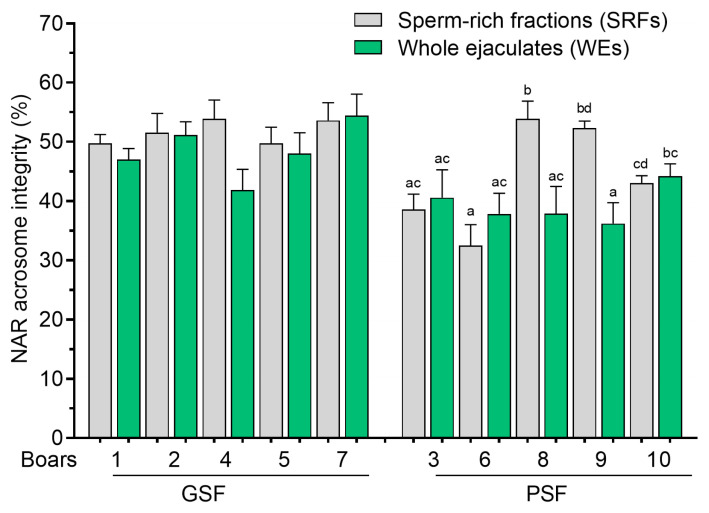
Normal apical ridge (NAR) acrosome integrity of frozen–thawed (FT) boar sperm. Within the freezability group, values with different letters (a, b, c, and d) are significant at *p* < 0.05. GSF—good semen freezability; PSF—poor semen freezability.

There were no differences (*p* > 0.05) in the PMI and NAR acrosome integrity of FT sperm between the SRFs and WEs or among boars of the GSF group (Figure 7 and Figure 8, respectively). However, two boars of the PSF group (Boar nos. 8 to 9) showed significantly higher (*p* < 0.05) PMI and NAR acrosome integrity in the SRFs compared with WEs (Figure 7 and Figure 8, respectively).

**Figure 9:**
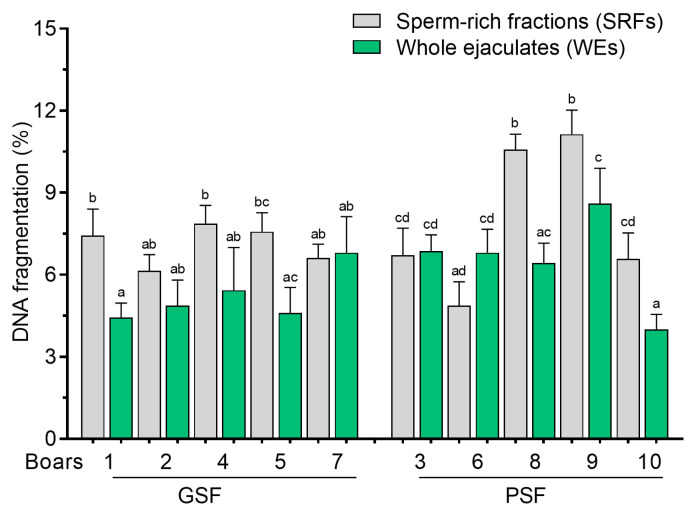
DNA fragmentation of frozen–thawed (FT) boar sperm. Within the freezability group, values with different letters (a, b, c, and d) are significant at *p* < 0.05. GSF—good semen freezability; PSF—poor semen freezability.

Wide variations in the DNA fragmentation of FT sperm were observed between the SRFs and WEs, particularly for the PSF group (Figure 9). It was observed that the DNA damage of FT sperm was significantly higher (*p* < 0.05) in the SRFs of three boars (Boar nos. 8 to 10) than in the PSF group (Figure 9). Furthermore, there was a tendency for lower DNA damage in FT sperm from the WEs of the GSF group (Figure 9).

### 3.4. Analyses of Correlation Heatmaps, Scatterplots, and Correlations

**Figure 10:**
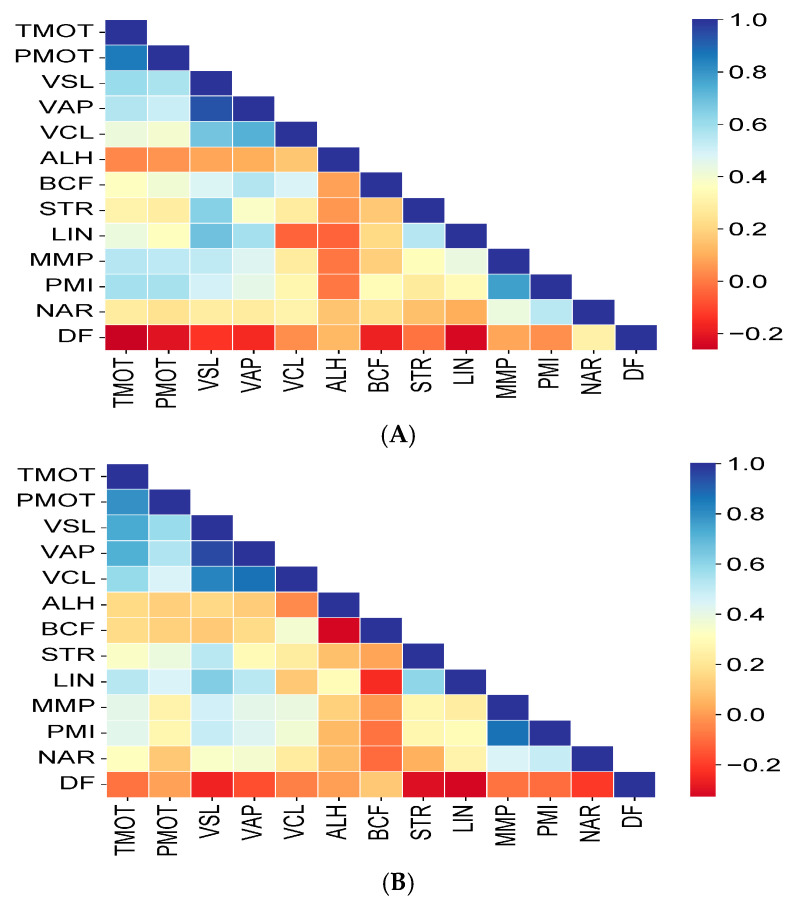
Correlation coefficient matrices of the relationships of the analyzed quality characteristics of frozen–thawed (FT) sperm from the (**A**) sperm-rich fractions (SRFs) and (**B**) whole ejaculates (WEs). The color scale represents the value of the correlation between two sperm variables as follows: blue highlights positive correlations, while dark brown highlights negative correlations. TMOT—total motility; PMOT—progressive motility; VSL—velocity straight line; VAP—velocity average path; VCL—velocity curvilinear; STR—straightness; LIN—linearity; ALH—amplitude of lateral head displacement; BCF—beat cross frequency; MMP—mitochondrial membrane potential; PMI—plasma membrane integrity; NAR—normal apical ridge acrosome integrity; DF—DNA fragmentation.

**Figure 11:**
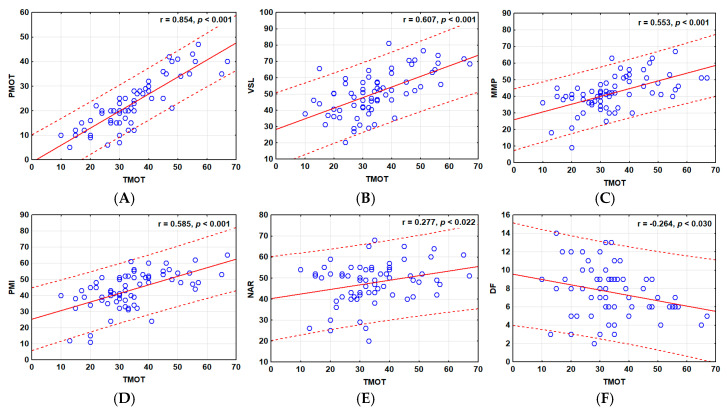
Scatter plot distributions of parameters of frozen–thawed (FT) sperm from the sperm-rich fractions (SRFs). (**A**) PMOT vs. TMOT, (**B**) VSL vs. TMOT, (**C**) MMP vs. TMOT, (**D**) PMI vs. TMOT (**E**) NAR vs. TMOT, (**F**) DF vs. TMOT. The red solid line represents the linear regression correlation, while the dotted red line represents the 95% confidence interval. The Pearson correlation coefficients are indicated in the plots. The Y-axis represents the different sperm parameters, while the X-axis represents total motility (TMOT). PMOT—progressive motility; VSL—velocity straight line; MMP—mitochondrial membrane potential; PMI—plasma membrane integrity; NAR—normal apical ridge acrosome integrity; DF—DNA fragmentation.

Most of the post-thaw sperm parameters of the SRFs were significantly positively correlated (*p* < 0.05), and these ranged from +0.854 to −0.264 (Figure 10A). No significant relationships (*p* > 0.05) were observed for the post-thaw ALH values, while post-thaw TMOT fragmentation was significantly negatively correlated (*p* < 0.05) with post-thaw DNA (Figure 10A and Figure 11F).

**Figure 12:**
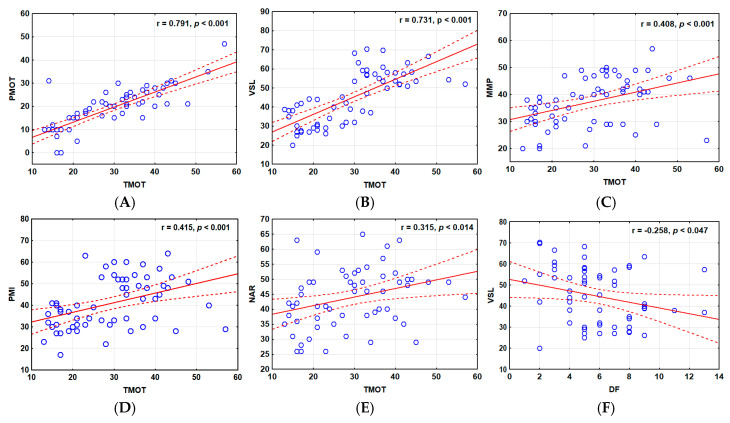
Scatter plots of parameters of frozen–thawed (FT) sperm from the whole ejaculates (WEs). (**A**) PMOT vs. TMOT, (**B**) VSL vs. TMOT, (**C**) MMP vs. TMOT, (**D**) PMI vs. TMOT (**E**) NAR vs. TMOT, (**F**) VSL vs. DF. The red solid line represents the linear regression correlation, while the dotted red line represents the 95% confidence interval. The Pearson correlation coefficients are indicated in the plots. The Y-axis represents the different sperm parameters, while the X-axis represents total motility (TMOT) or DNA fragmentation (DF). PMOT—progressive motility; VSL—velocity straight line; MMP—mitochondrial membrane potential; PMI—plasma membrane integrity; NAR—normal apical ridge acrosome integrity.

Similarly to the post-thaw semen of the SRFs, most of the relationships between the sperm parameters of the WEs were significantly positively correlated (*p* < 0.05) and ranged from +0.791 to −0.258 (Figure 10B). A weak significant relationship was observed between post-thaw ALH and LIN values (0.296, *p* < 0.05) (Figure 10B). Besides the negative correlation between VSL and DNA (Figure 10B and Figure 12F), weak negative significant relationships were also observed for ALH vs. BCF (−0.329, *p* < 0.01), STR vs. DNA (−0.304, *p* < 0.05), and LIN vs. DNA (−0.330, *p* < 0.01) (Figure 10B).

The scatter plot analyses confirmed the stronger linear relationships between the post-thaw TMOT with the analyzed parameters of the SRFs compared with those of the WEs (Figure 11 and Figure 12, respectively). The analyzed sperm parameters showed a constant trend with positive correlations, particularly for SRFs (Figure 11). Weak negative significant correlations were detected between post-thaw TMOT and DF of the GSF group (Figure 11F) and the post-thaw VSL values and DF of the PSF group (Figure 12F).

## 4. Discussion

Due to the complex biochemical composition of boar SP [16,27], quantitative variations within ejaculates and among individuals, or within different fractions, could have varying effects on the cryo-survival of sperm [17,18,19,36]. In this study, we analyzed the cryo-survival of sperm from the SRFs and WEs collected from boars with differing in semen freezability. Inter-boar variability and the composition of the SP are important factors that affect the functional and morphological structures of boar sperm membranes following freezing–thawing [6,7,19]. Our results showed that animal variability in the post-thaw quality characteristics was more marked in the poor freezability group, regardless of the sperm source. Even though we did not perform proteomic analysis of the SP of boars from the freezability groups, our findings suggest that the SP biochemical components are widely variable among the individual boars of the poor freezability group. Moreover, wide variations in the SP composition might be associated with marked differences in the structures of SP proteins and fatty acids, and their specific effects on the sperm functions among individual animals [16,37,38]. It is likely that such a phenomenon could explain the marked variability in freezability, which was particularly observed in the PSF group.

In this study, we hypothesized that, even though there were marked differences in the SRFs and WEs, particularly in the SP biochemical composition [16,27], there is reasonable post-thaw semen quality consistency between these two sperm sources of the GSF group. Although there are marked differences in the SP composition from the SRFs compared with the WEs, the GSF group did not show marked variations in post-thaw semen quality between the sperm sources. There was a similar trend for the PSF group. The clustering analysis, used to visualize the sample-to-sample distance (Figure 1), showed a minor outlier, particularly for one boar from the GSF group. The mean post-thaw TMOT was a reliable criterion used mainly to classify the boars [13,34,35]; it confirmed that most of the boars were grouped in the GSF or PSF group. PCA1, which is the main component of the sample variation, confirmed approximately 75% of the variance between the GSF and PSF groups. Furthermore, we generated the Pearson correlation matrix to examine the statistical significance between the post-thaw sperm parameters of the SRFs or WEs, regardless of the freezability group. This confirmed that associations were stronger for the analyzed post-thaw sperm parameters of the SRFs.

It is noteworthy that SRFs are commonly used in most studies based on the cryopreservation of boar semen. To our knowledge, little is known about the potential freezability of sperm from WEs. We observed marked differences between the SRFs and WEs in the TMOT, VSL, and VAP of FT sperm from three poor freezability boars. Regarding the good freezability group, only Boar no. 2 showed significant differences between its SRFs and WEs regarding post-thaw TMOT, PMOT, VSL, VAP, and LIN. Our findings show that the CASA-analyzed sperm parameters were positively correlated, regardless of the sperm source. In previous studies, it was reported that a combination of the CASA-analyzed sperm parameters, including VSL, VAP, ALH, and BCF, could be useful in estimating the fertility potential of boars [39,40]. It was demonstrated that approximately 24% of the variance in litter size due to boars being on commercial swine farms could be associated with differences in the CASA-analyzed sperm parameters [39]. In the current study, there were no significant relationships for post-thaw ALH values for SRFs, while ALH values correlated negatively with post-thaw BCF values for WEs, and at least two boars from the GSF group presented high post-thaw ALH values in the SRFs, alongside high post-thaw BCF values. According to Broekhuijse et al. [40], the ALH and BCF parameters are implicated in the sperm egg fertilization processes, and these are correlated with total number of piglets born (TNB) and the farrowing rate, respectively. In our study, there were no consistent changes in the CASA-analyzed motility patterns of FT sperm among the boars, regardless of the sperm source. In at least three of the analyzed post-thaw sperm parameters (VCL, PMI, and NAR acrosome integrity), there were no significant differences between the SRFs and WEs, or among the individual boars from the GSF group. However, there were consistent differences between the SRFs and WEs in MMP, PMI, NAR acrosome integrity, and DNA fragmentation of FT sperm from at least two boars (Boar no. 8 and Boar no. 9) of the PSF group. Accumulating evidence has indicated the beneficial effects of SP from the first portion of SRF (portion I [P1], 10 mL) on sperm cryo-survival [17,18,19]. According to Peña et al. [36], the freezability of some boar semen was better in the first portion of the SRF, post-SRF (portion II [P2]), or neither P1 nor P2. Besides the study of Peña et al. [36], the findings of other studies are consistent with the concept that cryo-survival is improved when sperm are exposed to specific portions of boar ejaculates during the freezing–thawing processes [17,18,19,23]. Moreover, it was demonstrated that prolonged storage of post-thaw semen in the presence of 50% SP improved sperm motility and viability [20]; even though some negative effects were observed, 50% SP could favor the overall performance of post-thaw semen [41]. According to Pini et al. [42], the SP is a highly beneficial supplement to FT semen, even though there are conflicting reports about the effect of SP in fertility studies. The inconsistent reports about the effects of SP on sperm cryo-survival have been attributed to several factors, including the methodology, SP protein concentrations, and semen processing procedure [42]. Moreover, the physiological role of boar SP components and their interactions with sperm membranes during freezing–thawing is not fully understood.

Notably, most of the proteins of the whole SP originate from boar vesicular glands, and most of them belong to the multifunctional spermadhesin lectin family of low molecular weight glycoproteins and the non-heparin binding porcine seminal plasma proteins I and II (the PSP-I/PSP-II heterodimer) [16,27,43,44]. Based on previous studies, these proteins could have varying effects on the cryo-survival of boar sperm [17,18,19]. Accordingly, several components, including protein substances, could attach to the sperm surface upon ejaculation and protect the sperm cells against damage following freezing–thawing [16,37]. Furthermore, a high bicarbonate ion concentration, and probably the lack of some sperm-coating proteins, have been suggested as explanation for the poor cryo-survival of sperm originating from the post-SRF [18,45]. In this study, we used the WEs, and we are not sure whether these factors are the main reason for the reduced cryo-survival of sperm from the poor freezability group. In our previous study, we demonstrated that some low molecular weight components of the SP affected sperm function, and that their removal by dialysis improved the cryo-survival of sperm [29]. Furthermore, we are not sure whether the low molecular weight components were more abundant in the WEs or SRFs, particularly from boars of the PSF group. Moreover, the protective effects of SP components depend on the degree of absorption of specific proteins present on the sperm membranes [46], which could vary between ejaculates and among boars [8,16,47]. We suggest that several factors might be involved in the regulation of the freezability of boar sperm. Besides the stabilizing protective effects of fibronectin-1 (FN-1) on the sperm structure following freezing–thawing [43,48], SP antioxidants, particularly superoxide dismutase (SOD), have been shown to enhance the cryo-tolerance of sperm from different fractions of boar ejaculate [49]. Recently, it has been demonstrated that increased antioxidant activity in the environment surrounding processed sperm might be related to a dynamic response that increases oxidative stress (OS), and which might promote AMP-activated protein kinase (AMPK), resulting in reduced apoptosis-induced sperm damage [50]. Moreover, differential metabolites in SP from good and poor freezability boars could be associated with the metabolic pathway of the AMPK-dependent regulation of sperm freezability [51]. It is noteworthy that the damage to sperm function following freezing–thawing is caused by multiple factors, including increased OS [41,42]. More recently, it has been confirmed that cryopreservation affects metabolic and redox proteins, thus compromising sperm-cryo-survival [11]. Evidence has confirmed that increased oxidative damage is a characteristic feature of ejaculates with poor freezability, thus making them more susceptible to cryo-induced damage [13].

Our results are in accordance with a previous study, indicating that the presence of SP from poor freezability boars compromises sperm cryo-survival [52]. As confirmed in our earlier studies, the SP of WEs tended to provide better DNA protection for FT sperm [21,30]. Also, other reports showed that the storage of FT semen in 10% and 50% SP reduced sperm chromatin alterations, but 50% SP exerted a more protective effect on the motility and chromatin integrity of FT sperm following prolonged storage [53]. Even though the mechanisms involved in the SP protection of FT sperm against chromatin alteration remain unknown [53], our results confirmed that the SP of WEs from the GSF group provided better protection to sperm DNA integrity following cryopreservation. However, less post-thaw DNA damage was not accompanied by higher sperm cryo-survival compared with other groups, suggesting the different responses of sperm cells to the freezing–thawing procedure. We suggest that a differential response among individual boars to the freezing–thawing procedure might be associated with the quantitative variations in the SP biochemical composition, thus affecting the cryo-tolerance of sperm.

The wide availability of freezability markers has enabled the selection of individual boars that produce ejaculates with good freezability [2,13,14,15,54]. Furthermore, the cryopreservation protocol used in this study shows that there were no consistent significant differences in the quality characteristics of FT sperm between the SRFs and WEs of either freezability group. Although we did not perform a proteomic evaluation of the SP of the SRFs and WEs from individual boars, we suggest that variations in the SP biochemical composition might affect the sperm cryo-survival.

## 5. Conclusions

In the current study, there were no consistent differences in the freezability potential of sperm between the SRFs and WEs from boars of the GSF group. Although we used a small animal population, our results have confirmed that boar variability is an important factor that affects sperm cryo-survival, regardless of the sperm source. Even though the precise role of SP components in cryo-survival of boar sperm has yet to been fully elucidated, marked differences in the quality characteristics of FT sperm from poor freezability boars might be associated with the quantitative variations in the SP biochemical components. We suggest that, under the conditions employed in this study, the freezing potential of sperm from the good freezability boars does not differ significantly between the SRFs and WEs; however, more research is warranted on a larger animal population.

## Figures and Tables

**Table 1:** ANOVA results showing the effects of boar variations and sperm source (SRF—sperm-rich fraction; WE—whole ejaculate) on the quality characteristics of sperm from the good semen freezability (GSF) group.

Sperm Parameters	Boar	Sperm Source	Boar × Sperm Source
	*p*-Value	*p*-Value	*p*-Value
Total motility (TMOT)	0.047	0.025	0.184
Progressive motility (PMOT)	0.057	0.004	0.407
Velocity straight line (VSL)	0.003	0.021	0.057
Velocity average path (VAP)	0.009	0.296	0.058
Velocity curvilinear (VCL)	0.584	0.931	0.582
Straightness (STR)	0.155	0.017	0.104
Linearity (LIN)	0.001	0.001	0.010
Amplitude of lateral head displacement (ALH)	0.015	0.003	0.210
Beat cross frequency (BCF)	0.001	0.006	0.001
Mitochondrial membrane potential (MMP)	0.066	0.019	0.235
Plasma membrane integrity (PMI)	0.097	0.142	0.953
Normal apical ridge (NAR) acrosome integrity	0.233	0.085	0.178
DNA fragmentation (DF)	0.673	0.002	0.452

The VAP, VCL, BCF, and ALH parameters were log-transferred prior to analysis. Significant at *p* < 0.05.

**Table 2:** ANOVA results showing the effects of boar variations and sperm source (SRF—sperm-rich fraction; WE—whole ejaculate) on the quality characteristics of sperm of the poor semen freezability (PSF) group.

Sperm Parameters	Boar	Sperm Source	Boar × Sperm Source
	*p*-Value	*p*-Value	*p*-Value
Total motility (TMOT)	0.353	0.001	0.033
Progressive motility (PMOT)	0.550	0.108	0.031
Velocity straight line (VSL)	0.004	0.001	0.045
Velocity average path (VAP)	0.004	0.001	0.199
Velocity curvilinear (VCL)	0.008	0.005	0.595
Straightness (STR)	0.875	0.095	0.108
Linearity (LIN)	0.014	0.064	0.387
Amplitude of lateral head displacement (ALH)	0.188	0.009	0.178
Beat cross frequency (BCF)	0.002	0.283	0.001
Mitochondrial membrane potential (MMP)	0.578	0.016	0.061
Plasma membrane integrity (PMI)	0.037	0.128	0.009
Normal apical ridge (NAR) acrosome integrity	0.020	0.033	0.002
DNA fragmentation (DF)	0.001	0.011	0.006

The VAP, VCL, BCF, and ALH parameters were log-transferred prior to analysis. Significant at *p* < 0.05.

## Data Availability

The datasets generated and analyzed during this study are contained within the article and Supplementary Materials.

## Supplementary Materials

The following supporting information can be downloaded at: https://www.mdpi.com/article/10.3390/cells14030212/s1. Supplementary Table S1: ANOVA results showing the effects of boar and sperm source, SRF (sperm-rich fraction), and whole ejaculate (WE) on the quality of fresh, pre-freeze (PF) semen. Supplementary Table S2: Quality characteristics of fresh, pre-freeze (PF) boar sperm from the sperm-rich fractions (SRFs) and whole ejaculates (WEs). GSF—good semen freezability; PSF—poor semen freezability.
