# Supplementary material for: Boar-to-Boar Variations in Quality Characteristics of Sperm from Different Ejaculates Following Freezing–Thawing
Source: Cells. 2025 Feb 2;14(3):212. doi: 10.3390/cells14030212 (PMC11817640; doi:10.3390/cells14030212)
Supplement: Supplementary file 1 [file cells-14-00212-s001.zip › cells-3356950-supplementary.pdf]

Supplementary Table S1. ANOVA results showing the effects of boar and sperm source, SRF (sperm-rich fraction) and whole ejaculate (WE) on the quality of fresh, pre-freeze (PF) semen

**Sperm parameters**	**Boar**	**Sperm source**	**Boar × Sperm source**
	***p*–Value**	***p*–Value**	***p*–Value**
Total motility (TMOT)	0.313	0.177	0.626
Progressive motility (PMOT)	0.072	0.637	0.932
Velocity straight line (VSL)	0.885	0.580	0.217
Velocity average path VAP)	0.317	0.572	0.518
Velocity curvilinear (VCL)	0.472	0.184	0.149
Straightness (STR)	0.742	0.303	0.519
Linearity (LIN)	0.065	0.105	0.933
Amplitude of lateral head displacement (ALH)	0.290	0.190	0.958
Beat cross frequency (BCF)	0.696	0.251	0.367
Mitochondrial membrane (MMP)	0.259	0.281	0.414
Plasma membrane integrity (PMI)	0.071	0.562	0.491
Normal apical ridge (NAR) acrosome integrity	0.799	0.535	0.541
DNA fragmentation (DF)	0.385	0.364	0.366

VSP, VAP, VCL, ALH and BCF parameters were log-transferred prior to analysis. Significant at $p < 0.05$

Supplementary Table S2. Quality characteristics of fresh pre-freeze (PF) boar sperm from the sperm-rich fractions (SRFs) and whole ejaculates (WEs). GSF – good semen freezability; PSF – poor semen freezability

	**SRFs (mean ± SEM)**		**WEs (mean ± SEM)**	
**Sperm parameters**	**GSF (*n* = 33)**	**PSF (n = 35)**	**GSF (n = 31)**	**PSF (*n* = 29)**
Total motility (TMOT, %)	87.82 ± 0.65	86.60 ± 0.68	87.97 ± 1.25	88.17 ± 0.70
Progressive motility (PMOT, %)	63.52 ± 0.98	65.06 ± 1.23	64.23 ± 1.22	65.34 ± 1.13
Velocity straight line (VSL, µm/s)	72.82 ± 1.38	71.29 ± 1.40	70.43 ± 1.48	74.50 ± 1.63
Velocity average path (VAP, µm/s)	90.38 ± 1.67	87.67 ± 1.75	88.95 ± 1.82	93.47 ± 2.28
Velocity curvilinear (VCL, µm/s)	117.74 ± 2.66	118.85 ± 2.48	110.07 ± 1.92	120.14 ± 3.11
Straightness (STR, %)	80.92 ± 1.33	81.52 ± 0.89	79.63 ± 1.41	80.13 ± 1.34
Linearity (LIN, %)	62.39 ± 1.26	60.41 ± 1.07	64.21 ± 1.12	62.42 ± 1.16
Amplitude of lateral head displacement (ALH, µm)	7.22 ± 0.26	7.34 ± 0.19	6.97 ± 0.32	6.98 ± 0.26
Beat cross frequency (BCF, Hz)	31.44 ± 0.82	29.54 ± 0.77	31.36 ± 1.08	29.58 ± 0.83
Mitochondrial membrane potential (%)	88.68 ± 0.65	89.74 ± 0.52	86.87 ± 0.59	88.04 ± 0.67
Plasma membrane integrity (PMI, %)	88.30 ± 0.61	87.29 ± 0.73	85.35 ± 0.62	84.56 ± 1.65
Intact acrosome integrity (%)	92.84 ± 0.71	91.97 ± 0.56	92.16 ± 0.61	93.90 ± 0.74
DNA fragmentation (DF, %)	2.20 ± 0.29	2.12 ± 0.27	1.84 ± 0.32	1.68 ± 0.57

Differences among the parameters were analyzed with the Tukey HSD post *hoc test*
